# Draft genome sequences of *Mycobacterium caprae* strains linked to human tuberculosis in Canada

**DOI:** 10.1128/mra.00234-25

**Published:** 2025-05-20

**Authors:** Md Rashedul Islam, Pierre-Marie Akochy, Gregory J. Tyrrell, Debra Janella, Meenu K. Sharma, Hafid Soualhine

**Affiliations:** 1 National Reference Centre for Mycobacteriology, National Microbiology Laboratory, Public Health Agency of Canada 85072 , Winnipeg, Manitoba, Canada; 2 Institut National de Sante Publique, Laboratoire de Santé publique du Québec, Montreal, Québec, Canada; 3 Alberta Public Health Laboratory, Alberta Health Services 3146, Edmonton, Alberta, Canada; 4 Department of Medical Microbiology and Infectious Diseases, University of Manitoba 574854, Winnipeg, Manitoba, Canada; Rochester Institute of Technology, Rochester, New York, USA

**Keywords:** *Mycobacterium caprae*, human tuberculosis, genome sequence, diagnostics, drug susceptibility testing

## Abstract

*Mycobacterium caprae* is a causative agent of tuberculosis that affects both humans and animals. Here, we present the draft genome sequences of two *M. caprae* strains. These genome sequences may improve understanding of *M. caprae* epidemiology and support diagnostic development.

## ANNOUNCEMENT

*Mycobacterium caprae*, first proposed as *M. tuberculosis* subsp. *caprae* in 1999 (1) and later elevated to the species rank (2), causes tuberculosis in livestock, wildlife animals, and humans (3–7). *M. caprae* has been described as the animal-adapted lineage “La2” by recent nomenclature (8). Presently, a small number of human-originated *M. caprae* genome sequences are available, and thus, the epidemiology of this organism is still poorly understood. In the present study, two clinical *M. caprae* strains 300103 and 800347 were isolated from peritoneal fluid and sputum samples of patients who were sick with tuberculosis in the Canadian provinces of Quebec in 2002 and Alberta in 2008, respectively. The provincial laboratories grew the original cultures 300103 and 800347 aerobically at 37°C onto Löwenstein-Jensen slant for 2 weeks and in BACTEC tube in the BD BACTEC 460 System for a week, respectively. The cultures were forwarded to the National Reference Centre for Mycobacteriology for identification. The identification was confirmed by the presence of a special combination of *pncA*, *katG,* and *gyrA* gene mutations as described by Aranaz et al. (2) and other unique mutations in the *gyrB* (2, 9) and *LeuS* genes (9) specific to *M. caprae*. Phenotypic drug susceptibility testing (DST) for the first-line drugs (i.e*.,* isoniazid, rifampicin, ethambutol, and pyrazinamide) was performed using the BACTEC 460 System. Genomic DNA was extracted using the InstaGene Matrix (9) comprising 6% (w/v) Chelex resin for PCR-ready DNA purification (Bio-Rad#7326030; California, United States) and sequenced using an Illumina MiSeq platform (Illumina Inc.), with the Illumina DNA Prep Kit to generate 2 × 150 bp. Raw sequencing reads were quality checked using FastQC (10) and SMALT (https://www.sanger.ac.uk/tool/smalt-0/) in a Galaxy (11) workflow. The reads were processed in the IRIDA (12) by Shovill v.1.0.4, which used SPAdes v.3.14.1 for *de novo* assembly (13) and QUAST for assembly assessment (14). The genome annotation was carried out by the National Center for Biotechnology Information (NCBI) Prokaryotic Genome Annotation Pipeline 2024-07-18.build7555 (15). CheckM v.1.2.3, incorporated in the PGAP, was used to assess the quality of the genomes. Mykrobe v.0.10.0 (16) and TBProfiler v.6.4.0 (17) were employed for predicting antimicrobial resistance. Default parameters were used for all software unless otherwise mentioned.

The assembly and annotations statistics are shown in Table 1. The genomes of *M. caprae* strains 300103 and 800347 contain 4,101 and 4,102 predicted coding DNA sequences, respectively, with a high G+C content (65.5%). Each strain has 45 tRNAs and three complete rRNAs (one copy of each 5S, 16S, and 23S). FastANI (v1.33) (18) whole-genome comparison of 300103 and 800347 with a reference *M. caprae* ATCC BAA-824 (2, 19) generated an average nucleotide identity of 99.93% and 99.94%, respectively. The strains were predicted to be fully susceptible to pyrazinamide, isoniazid, rifampicin, and ethambutol. These results were concordant with phenotypic DST.

**TABLE 1:** Genomic features of the *Mycobacterium caprae* strains

Strain name	No. of reads	Coverage (×)	Assembly size (bp)	N50 (bp)	No. of contigs	No. of CDSs^*a*^	No. of genes	No. of rRNAs	No. of tRNAs	Genome completeness (%) - CheckM	Genome contamination (%) - CheckM	Genome accession no.
300103	1,514,982	68.05	4,347,424	136,136	79	4,101	4,152	3	45	95.25	0.37	JBKGTX000000000
800347	2,870,356	179.55	4,311,143	104,531	111	4,102	4,153	3	45	94.73	0.37	JBLBUU000000000

^*a*^ CDSs, coding DNA sequences.

## Data Availability

The raw reads and assembled genome sequences of *M. caprae* strains 300103 and 800347 have been deposited in the NCBI database under BioProject PRJNA1204945. The accession codes for raw and assembled data are SRR31884848 and SRR31884847 and JBKGTX000000000 and JBLBUU000000000.
